# Accuracy of the plethysmographic variation index as a predictor of fluid responsiveness after cardiac surgery

**DOI:** 10.1186/cc13342

**Published:** 2014-03-17

**Authors:** H Merdji, P Biston, M Piagnerelli

**Affiliations:** 1 CHU de Reims, France; 2 CHU de Charleroi, Belgium

## Introduction

Recent studies showed that the plethysmographic variability index (PVI), a dynamic index resulting from cardiopulmonary interactions, could predict fluid responsiveness (FR) in mechanically ventilated patients during general anesthesia and in the ICU [1],[2]. In this study, we aimed to compare, in patients after cardiac surgery, the clinical utility of the PVI versus traditional statics and dynamics indices to predict FR.

## Methods

We prospectively enrolled 52 consecutive adult patients in sinus rhythm and mechanically ventilated (tidal volume of 8 (7.5 to 8.5) ml/kg ideal body weight) admitted to the ICU of CHU de Charleroi (Belgium) after scheduled cardiac surgery. Before and after fluid challenge (FC), we measured: heart rate (HR), BP, PVI (Masimo Corporation, Irvine, CA, USA) and PPV. PAOP and cardiac index (CI) were estimated by Swan-Ganz catheter (Edwards Lifesciences LLC, Irvine, CA, USA). Patients with an increase in CI of at least 15% after FC and subsequently were considered responders (R). Continuous variables were analyzed with Mann-Whitney *U *tests. Categoric variables were analyzed with the Fisher's exact test. Correlations were assessed by Spearman's correlation. The discriminatory ability of each haemodynamic parameter to determine FR was assessed by area under the receiver operating characteristic curve (AUC), between R and nonresponders (NR). *P *< 0.05 was considered statistically significant.

## Results

Twenty-five (48%) patients were classified as R. Before FC, R and NR patients were comparable except for HR (91 (83 to 108) for R vs. 80 beats/minute (72 to 89) for NR; *P *= 0.009), PVI (18 (12 to 26) for R vs. 12% (9 to 15) for NR; *P *= 0.003), PAOP (8 (6 to 9) for R vs. 11 mmHg (8 to 12) for NR; *P *= 0.001), and PPV (13 (8 to 19) for R vs. 10% (4 to 13) for NR; *P *= 0.086). PVI and delta PP before FC were correlated (*r *= 0.53, *P *< 0.0001). AUC showed a better prediction of FR for PVI (AUC = 0. 74. *P *< 0.001) than for PPV (AUC = 0.64, *P *= 0.05). Nevertheless, the sensitivity (Se) and specificity (Sp) of both indices were low: for a PPV over 13%, Se = 48% (0.301 to 0.665), and Sp = 82% (0.627 to 0.921); for a PVI over 12%, Se = 68% (0.482 to 0.828) and Sp = 69% (0.498 to 0.835). As for PAOP, increasing values are associated with NR (AUC = 0.22, *P *< 0.0001).

## Conclusion

The PVI appears to be useful to predict FR in mechanically ventilated patients after cardiac surgery. However, Se and Sp remain low in the type of patients with relative euvolemia and cardiac dysfunction.
